# EHNet: Efficient Hybrid Network with Dual Attention for Image Deblurring

**DOI:** 10.3390/s24206545

**Published:** 2024-10-10

**Authors:** Quoc-Thien Ho, Minh-Thien Duong, Seongsoo Lee, Min-Cheol Hong

**Affiliations:** 1 Department of Information and Telecommunication Engineering, Soongsil University, Seoul 06978, Republic of Korea; hoquocthiendl@soongsil.ac.kr; 2 Department of Automatic Control, Ho Chi Minh City University of Technology and Education, Ho Chi Minh City 70000, Vietnam; minhthien@hcmute.edu.vn; 3 Department of Intelligent Semiconductor, Soongsil University, Seoul 06978, Republic of Korea; sslee@ssu.ac.kr; 4 School of Electronic Engineering, Soongsil University, Seoul 06978, Republic of Korea

**Keywords:** convolution neural networks, dual attention module, hybrid architecture, image deblurring, motion blur, Transformer

## Abstract

The motion of an object or camera platform makes the acquired image blurred. This degradation is a major reason to obtain a poor-quality image from an imaging sensor. Therefore, developing an efficient deep-learning-based image processing method to remove the blur artifact is desirable. Deep learning has recently demonstrated significant efficacy in image deblurring, primarily through convolutional neural networks (CNNs) and Transformers. However, the limited receptive fields of CNNs restrict their ability to capture long-range structural dependencies. In contrast, Transformers excel at modeling these dependencies, but they are computationally expensive for high-resolution inputs and lack the appropriate inductive bias. To overcome these challenges, we propose an Efficient Hybrid Network (EHNet) that employs CNN encoders for local feature extraction and Transformer decoders with a dual-attention module to capture spatial and channel-wise dependencies. This synergy facilitates the acquisition of rich contextual information for high-quality image deblurring. Additionally, we introduce the Simple Feature-Embedding Module (SFEM) to replace the pointwise and depthwise convolutions to generate simplified embedding features in the self-attention mechanism. This innovation substantially reduces computational complexity and memory usage while maintaining overall performance. Finally, through comprehensive experiments, our compact model yields promising quantitative and qualitative results for image deblurring on various benchmark datasets.

## 1. Introduction

Motion blur is a common image degradation phenomenon caused by camera shaking or object movement during image capture, which reduces the image quality and impairs the performance of computer vision algorithms [1]. Despite high-speed cameras providing a hardware-based solution to capture sharper images, they often produce underexposed photos. Therefore, software-based image deblurring is explored to restore high-quality images. The major goal of these algorithms is to recover sharp details and the underlying structure of a clear image from its blurry counterpart [2,3]. Although it is undeniable that these methods have obtained promising results, some limitations remain that are worth investigating.

Traditional image deblurring methods rely on pre-defined priors to guide the deblurring process [4,5,6]. These methods typically assume uniform blurring across the entire image, thereby simplifying the deblurring problem and failing to capture the complexities of real-world scenarios. Although some studies have explored priors that attribute non-uniform blur [7,8], the dynamic nature of real-world scenes with various moving objects still poses challenges in achieving optimal deblurring quality.

Deep learning has been predominant in computer vision for an extended period, encompassing low-level vision tasks [9,10,11,12]. Convolutional neural networks (CNNs) [13] have significantly improved performance owing to their capacity to learn intricate representations and extract spatially local features, making them particularly effective in image deblurring. Early CNN-based image deblurring methods typically adopted a two-stage approach with the CNN stage estimating the blur kernel, followed by the deconvolution stage [14,15]. DeepDeblur [16] pioneered the use of an end-to-end CNN to train a deblurring model directly from blurry–sharp image pairs. It laid the foundation for CNN-based deblurring methods to enhance performance substantially [17,18,19,20,21,22,23,24]. A major problem of conventional CNNs is their restricted receptive fields. Due to the ineffective long-range dependency structure, CNN-based methods struggle to address non-uniform blur at local and global scales [25]. Despite efforts to increase the number of convolution layers or expand kernel sizes to enlarge the receptive fields, these manners commonly result in a significantly higher computational cost and model size. Furthermore, researchers have investigated a diverse set of hierarchical CNN architectures [26,27,28,29,30,31,32,33,34] aimed at improving the effectiveness of image deblurring methods. Another limitation of conventional CNNs is their reliance on fixed shared weights [35], which restricts their adaptability to extremely dynamic scenes with multiple moving objects. To address these limitations above, Transformer-based techniques have recently been studied by incorporating self-attention mechanisms that can capture long-range dependency structures and dynamically adapt to various inputs. Most representatively, Transformer-based models have shown promising results for image deblurring tasks [36,37,38,39]. However, their complexity and memory demands escalate dramatically when processing high-resolution inputs. One venial reason for this issue is the scaled dot-product operation related to the input size. In addition, the embedding feature extraction in each self-attention module relies on pointwise convolutions. The computation cost of these operations increases proportionally with the number of channels. It becomes extremely high in multi-scale architectures, where the number of channels doubles after each downsampling step. Another limitation of vanilla Transformers is the lack of the right inductive bias [40]. In order to address this issue, many studies [41,42,43,44] have proposed hybrid networks that combine CNNs and Transformers to fuse local and long-range features to enhance deblurring quality. However, their use of fixed window sizes in embedding features of Transformer modules limits the flexibility in capturing the diversity scales of the blur patterns.

In brief, the previous deep-learning models for image deblurring exhibit several limitations: (1) CNNs suffer from restricted receptive fields and the inflexibility of fixed shared weights. (2) Vanilla Transformers lack the right inductive bias, and their embedding tokens are generated using fixed-size window features that limit their ability to capture diverse blur patterns. (3) Many existing image deblurring methods often involve large model sizes and high computational complexity.

**Figure 1:**
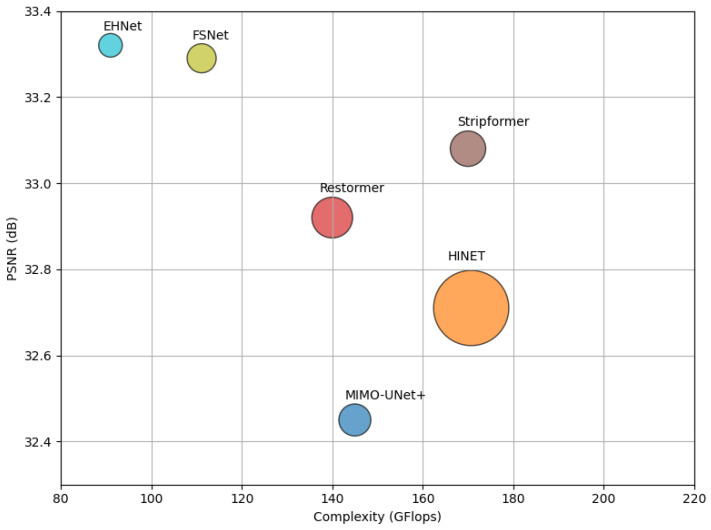
Comparison of the proposed Efficient Hybrid Network (EHNet) and the state-of-the-art deep-learning methods on the GoPro dataset in terms of peak signal-to-noise ratio (PSNR), complexity, and network parameters. In that, the circle size indicates the size of the methods.

To address these limitations, we propose the Efficient Hybrid Network (EHNet) for image deblurring. EHNet combines CNN encoders to extract local features with Transformer decoders capable of capturing long-range dependencies to model contextual relationships across the entire image. This hybrid design leverages the complementary strengths of both architectures to address the limited receptive fields of CNNs and the lack of right inductive bias in Transformers. Furthermore, we introduce two attention modules in the Transformer decoder. More specifically, the Multi-Head Transposed Attention (MHTA) module refines the features by explicitly modeling channel dependencies. Meanwhile, the Multi-Window Self-Attention (MWSA) module enables the network to analyze diverse blur patterns by focusing on long-range dependencies across different spatial window scales. Through the fusion of outputs from the MHTA and MWSA modules, the EHNet extracts comprehensive features to improve the image deblurring performance. In addition, we propose the Simple Feature-Embedding Module (SFEM) as a replacement for the pointwise and depthwise convolutions to generate simplified embedding features in the self-attention mechanism. Thereby, the computational costs and model size can effectively be reduced without sacrificing the overall performance of our network. The efficiency of the proposed EHNet is illustrated in Figure 1.

In summary, this work introduces several key contributions:

- The EHNet leverages a combination of CNN encoders and Transformer decoders to achieve efficient image deblurring.
- Transformer Blocks are improved to address the limitations of previous methods. The proposed blocks include the following: (1) The MHTA refines features by modeling channel dependencies. (2) The MWSA captures blur patterns at various spatial scales. (3) The SFEM generates simplified embedding features in each self-attention module.
- Our comprehensive evaluation reveals that EHNet outperforms existing state-of-the-art deep-learning methods in image deblurring. This superior performance is achieved through a compact model size and reduced computational demands.

The remainder of this paper is organized as follows. Section 2 reviews the existing deep-learning approaches for image deblurring, focusing on end-to-end CNN-based and Transformer-based networks. Section 3 describes the proposed method for building an efficient image deblurring network. Section 4 analyzes the experimental results and Section 5 concludes the paper.

## 2. Related Work

In addition to traditional methods [4,5,6,7,8], deep learning offers innovative approaches to image deblurring, with CNNs and Transformers being the two leading techniques in this field. This section discusses their capabilities and how they can restore clear images from blurry ones.

### 2.1. CNN-Based Image Deblurring

Recently, CNNs have demonstrated significant advantages in image deblurring. The pioneering work of DeepDeblur [16] introduced an end-to-end deep-learning framework comprising an encoder–decoder architecture that utilizes pairs of blurry and sharp images to train the deblurring model. Building on this foundation, several deblurring models trained solely with pixel loss conventionally improve the quantitative performance, but lead to over-smoothing restoration images [21,22]. To address this issue, generative adversarial networks (GANs) have been employed to produce more realistic images [18,19,20]; however, they can also introduce undesired artifacts. Furthermore, hierarchical multi-scale architectures have been studied to incorporate the information of scales and stages; the scale-recurrent network (SRN) [17] processed the blurry input image at smaller resolutions and progressively refined the output to larger resolutions. The Deep-Stacked Multi-Patch Hierarchical Network (DMPHN) [26] considered that using more scales does not improve performance and introduced a strategy to divide the blurry image into multiple patches for processing. Cho et al. [27] investigated coarse-to-fine features and proposed a multi-input multi-output U-Net (MIMO-UNet) architecture to reduce the overall computational cost. In further attempts to explore the features of different stages, a multi-stage progressive image restoration architecture called MPRNet [28] proposed a cross-stage feature fusion and introduced a supervised attention module to refine the output features. The half-instance normalization network (HINet) [29] mitigates the influence of batch dimensions and preserves scale information by incorporating a branch with instance normalization. In addition, the blur-aware attention network (BANet) [31] proposed blur-aware attention for disentangling blur patterns of different magnitudes or orientations to solve dynamic scene deblurring.

Despite the significant success of CNNs in image deblurring, their inherent limitations hinder performance improvements. The constrained receptive fields of CNNs fail to capture long-range dependencies. Although adding more convolution layers can alleviate this issue, it also significantly increases the complexity of the network. Furthermore, the fixed shared weights [35] of CNNs hamper their ability to adapt to dynamic scenes and non-uniform blur effects.

### 2.2. Transformer-Based Image Deblurring

Transformers with self-attention mechanisms have become powerful tools for high-level vision tasks and demonstrate state-of-the-art performance [45,46]. This approach offers advantages over CNNs, particularly in its ability to capture long-range dependency structures to model contextual relationships across an entire image. As the pioneer applied Transformer to low-level vision, Uformer [36] presented the U-shaped Transformer architecture for various image restoration tasks including image deblurring. Restormer [37] proposed Multi-Head Transposed Attention for channel-wise attention, which can model long-range pixel interactions while remaining applicable to large images. Zhao et al. [38] proposed an efficient Transformer to capture the superpixel-wise dependency and then transfer it to each pixel. In order to consider factors such as locality, non-locality, and cross-scale aggregation, the study [39] introduced a cross-scale k-NN transformer to group similar patches in the whole image and conduct local attention. In addition, Xu et al. [47] developed a bidirectional Transformer to consider long-range dependency in both temporal and spatial for video deblurring.

Although Transformer-based models have demonstrated significant potential for image deblurring, they face several limitations. The primary challenge lies in the extremely high complexity and large model size when dealing with high-resolution input images. Furthermore, the absence of the right inductive bias in Transformers [40] can deter the extraction of desirable local contexts in images. This issue may constrain their ability to achieve optimal spatially localized deblurring. Therefore, the hybrid architectures of CNN and the Transformers are proposed to fuse the local and long-range information of images [41,42,43,44]. More representatively, Stripformer [41] introduced CNN encoders and employed Transformers with horizontal and vertical strip attention in decoders to capture global blurred patterns with different orientations. Considering the computational burden of Transformers, Sharpformer [42] employed the Transformer only at the final scale of the CNN model to minimize the network complexity. The study [43] considered the hybrid multiscale architecture that combines CNN and Transformer and proposed a feature modulation network to alleviate the disadvantages of CNN sub-networks that lack input content adaptation in image deblurring. Furthermore, the authors of [44] proposed multi-scale architecture consisting of CNN and Transformer to recover the edge contours and texture of the images more clearly.

## 3. Proposed Method

In this section, the architecture of the proposed network is discussed. We then explore the proposed components and loss function used to train the model.

### 3.1. Overall Architecture

**Figure 2:**
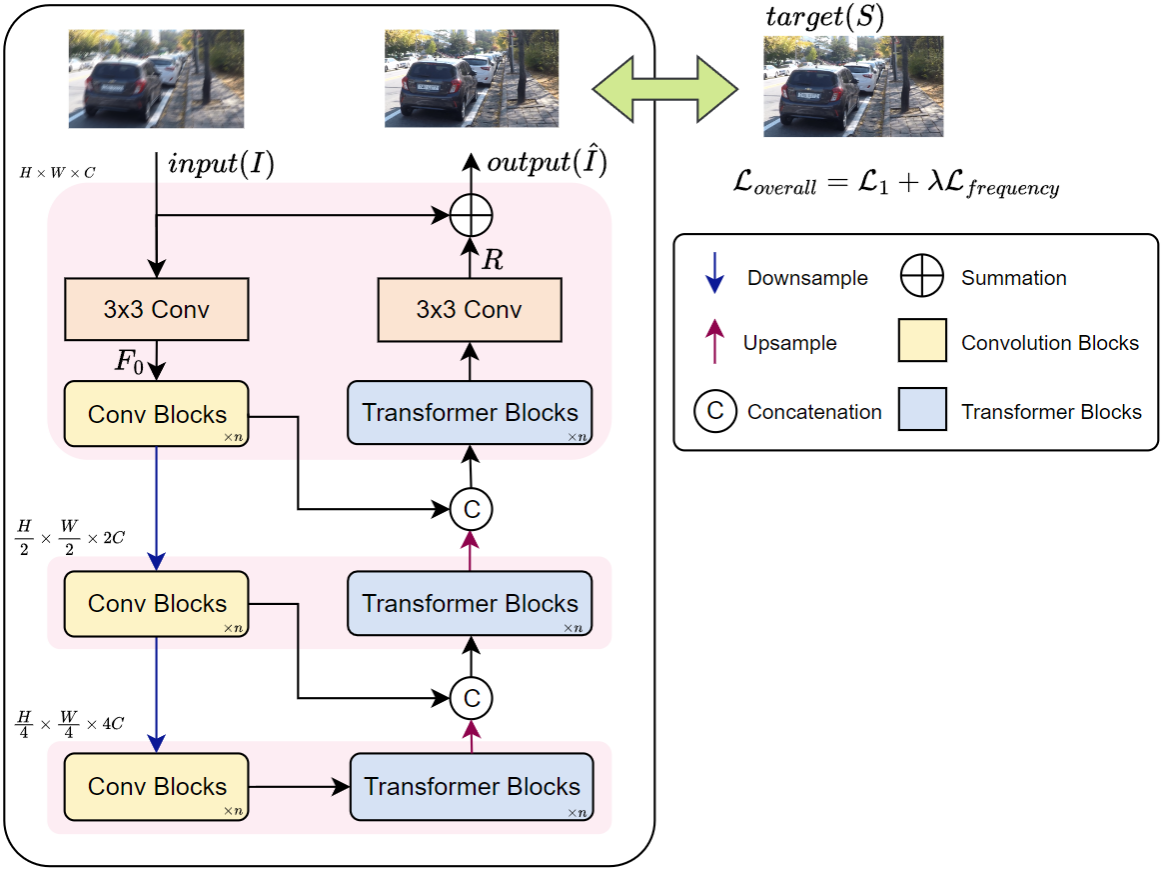
The overall architecture of our proposed EHNet for image deblurring.

This study introduces EHNet, an Efficient Hybrid Network designed for image deblurring. The EHNet leverages the complementary strengths of CNNs and Transformers to achieve high-quality deblurring results. The CNN encoders extract local features and pass them to the corresponding Transformer decoders at each scale. This synergy between the strong inductive bias of CNNs and the capability of Transformers to model long-range dependencies allows EHNet to adapt to diverse blur scales. Thereby, the robustness of the network can be enhanced. The overall architecture of EHNet is illustrated in Figure 2.

The EHNet employs a multi-scale encoder–decoder architecture for image deblurring. It takes a blurry image $I\in R^{H\times W\times 3}$ as input; where H,W represent the image height and width, and 3 denotes the R, G, B channels. The initial process applies a 3×3 convolution to extract shallow features $F_{0}\in R^{H\times W\times C}$. The number of channels *C* in $F_{0}$ defines the feature depth during the first stage. Following the initial convolution, the network utilizes a three-scale structure to perform finer deblurring. Each scale of EHNet includes encoder and decoder blocks. They leverage pointwise and depthwise convolutions [48] instead of standard convolution operations to enhance the efficiency of the network. The encoder at each scale incorporates a dedicated downsampling branch to progressively reduce the spatial dimensions by half and double the number of channels relative to the preceding input feature map. The encoder function at each scale *i* can be formally expressed as:

(1) $$F_{i}=E_{i}\left(\cdot \right)=\left\{\begin{array}{cc} C_{i}\left(F_{0}\right), & i=1\\ C_{i}\left(F_{i-1}↓\right), & i=2,3 \end{array}\right.,$$

where $E_{i}\left(\cdot \right)$, $C_{i}\left(\cdot \right)$, and $F_{i}$ denote the encoder, Convolution Blocks, and output feature of the encoder at scale *i*, respectively. The downsampling operation (↓) signifies the downscaling via bilinear interpolation.

Following feature extraction by the encoder at each scale (i), the resulting feature maps are passed through the corresponding decoder, which can be expressed as follows:

(2) $$F_{i}^{\prime }=D_{i}\left(\cdot \right)=\left\{\begin{array}{cc} T_{i}\left(F_{i+1}^{\prime }↑⊕F_{i}\right), & i=1,2\\ T_{i}\left(F_{i}\right), & i=3 \end{array}\right.,$$

where $D_{i}\left(\cdot \right),T_{i}\left(\cdot \right),$ and $F_{i}^{\prime }$ denote the decoder, Transformer Blocks, and output features of the decoder at scale *i*, respectively. The symbol ⊕ represents the concatenation operation and the upward arrow (↑) denotes the upsampling operation via the bilinear interpolation. During upsampling, the spatial resolution of the feature map typically expands by a factor of 2, whereas the number of channels is halved. At all scales except the last one, the output feature of encoder $F_{i}$ is concatenated with the upsampled feature $F_{i+1}^{\prime }$ coming from the higher-level scale decoder (i+1) and then provides these features to the Transformer decoder at the current scale, denoted as $T_{i}\left(\cdot \right)$. This process progressively enhances the coarse features from high scale to fine features of low scale. At the highest scale (i=3), the decoder directly processes the features received from the corresponding encoder output. Moreover, the Transformer decoders employ a dual-attention mechanism, combining both spatial and channel self-attention. This integration of attention mechanisms enables the Transformer to aggregate essential features for high-quality image deblurring.

Following the last decoder block, a 3×3 convolution is applied to the feature maps to obtain a residual image $R\in R^{H\times W\times 3}$. Finally, the restoration images can be expressed as:

(3) $$\hat{I}=I+R=I+N\left(I\right),$$

where $\hat{I}$ and N(·) denote the restored image and encoder–decoder of EHNet, respectively.

### 3.2. Convolution Blocks

**Figure 3:**
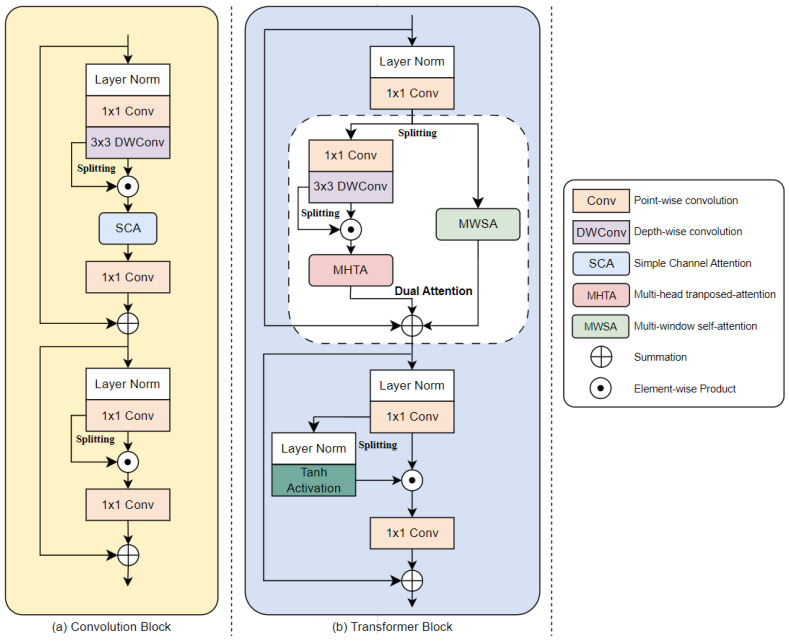
The detailed structure of the main components in the proposed network.

Hierarchical multi-scale architectures leverage CNNs, but their limited receptive fields constrain the capture of long-range dependency structures. Several previous methods expand the convolutional kernel sizes or increase the number of network layers, resulting in significant computational costs and memory usage. The EHNet mitigates the overall complexity by replacing standard convolution operations with pointwise and depthwise convolutions [48] in all the Convolution Blocks. Inspired by recent works [32,37,49], our proposed Convolution Block adopts well-established components such as layer normalization [50], simple channel attention (SCA) [32], and skip connections [51]. The detailed structure of the Convolution Blocks is shown in Figure 3a.

### 3.3. Transformer Blocks

The core of our Transformer is the dual-attention module, which incorporates self-attention mechanisms in the channel and spatial domains, as shown in Figure 3b. Initially, both attention modules employ SFEM to extract simplified features. Subsequently, we introduce an MHTA module to model feature dependencies in the channel domain and the MWSA module to capture the relationships between spatial elements at different scales. Integrating these attention mechanisms aggregates the essential information for image deblurring.

#### 3.3.1. Simple Feature-Embedding Module (SFEM)

**Figure 4:**
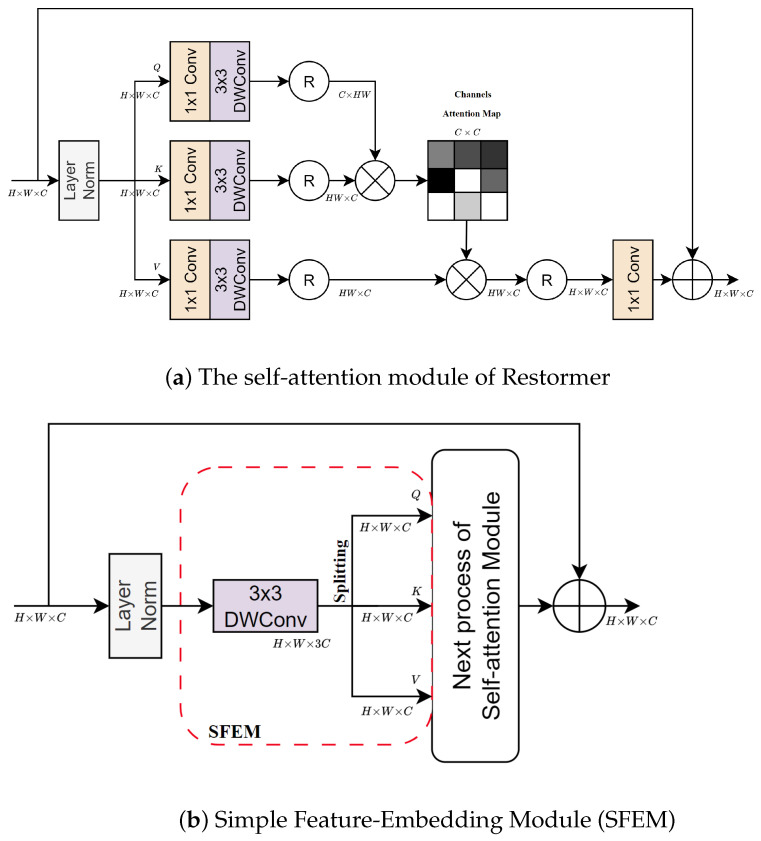
The feature extraction process in self-attention modules. SFEM extracts simple local features via single depthwise convolution, splits them into branches, and passes them to the next self-attention step.

In addition to the standard scaled dot-product operator, pointwise convolution operations used to extract the embedding features in the self-attention module also cause a computational burden. Although pointwise convolutions may seem computationally efficient at $C^{2}HW$ per operation with $C^{2}$ parameters, their dependence on the number of channels presents a significant challenge. This issue is particularly problematic in multi-scale architectures, where the number of channels is doubled after each downsampling step. The computational burden is further amplified when pointwise convolution operations are used multiple times within each self-attention module as illustrated in Figure 4a.

To mitigate the computational expense associated with pointwise convolutions in the Transformer decoder of the EHNet, we propose an alternative method utilizing depthwise convolutions to extract local features and split the output into three branches in the self-attention module, which is called the Simple Feature-Embedding Module (SFEM). The SFEM is presented in Figure 4b. The depthwise convolution operation is only applied to one filter per input channel; thus, the computational cost and memory usage are significantly reduced to $CK^{2}HW$ and $CK^{2}$ (given $K^{2}<<C$), respectively. Finally, SFEM generates simplified embedding features used as the input for the scaled dot-product operation, which is especially efficient at higher scales of multi-scale networks characterized by larger channel numbers. Formally, the SFEM can be expressed as:

(4) $$Q,K,V=SFEM\left(X\right)=Split(W_{d}\left(X\right)),$$

where SFEM(·) denotes the Simple Feature-Embedding Module and $W_{d}\left(\cdot \right)$ represents the depthwise convolution operation. Additionally, Split(·) denotes the splitting operation.

#### 3.3.2. Multi-Head Transposed Attention (MHTA)

We expand on the achievements of Restormer [37], which employs self-attention in the channel domain to model long-range dependencies and interactions among channels. In particular, the MHTA is proposed, utilizing the SFEM to generate simplified embedding features before introducing them into the scaled dot-product operation of self-attention in the channel domain.

**Figure 5:**
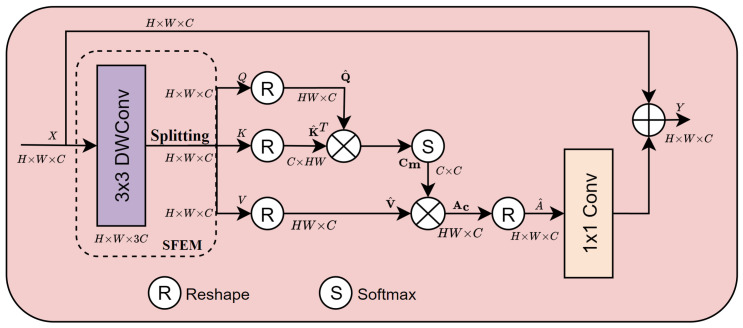
The structure of Multi-Head Transposed Attention (MHTA) module.

The MHTA structure is illustrated in Figure 5. First, the MHTA passes the input feature $X\in R^{H\times W\times C}$ through the SFEM to generate the embedding features query, key, value $Q,K,V\in R^{H\times W\times C}$ as follows:

(5) Q,K,V=SFEM(X).

The query, key, and value are then reshaped to obtain linear projection matrices $\hat{Q},\hat{K},\hat{V}\in R^{HW\times C}$. The feature attention $A_{c}\in R^{HW\times C}$ is obtained by calculating:

(6) $$A_{c}=softmax\left(\frac{\hat{Q}{\hat{K}}^{T}}{\alpha }\right)\hat{V},$$

where α denotes the learnable scaling parameter used in the softmax function. The attention map $C_{m}\in R^{C\times C}$ representing the cross-channel interaction is achieved by the scaled dot-product of the query ($\hat{Q}$) and transposed key (${\hat{K}}^{T}$). Then, we reshape feature attention $A_{c}$ back to the original input size to obtain feature $\hat{A}\in R^{H\times W\times C}$. The final output of the MHTA module is calculated as:

(7) $$Y=W_{p}\left(\hat{A}\right)+X,$$

where *X* and *Y* are the input and output feature maps, respectively, and $W_{p}\left(\cdot \right)$ denotes the pointwise convolution. Similar to [45], we divide the number of channels into multiple heads (n=8) and learn attention maps in parallel.

#### 3.3.3. Multi-Window Self-Attention (MWSA)

**Figure 6:**
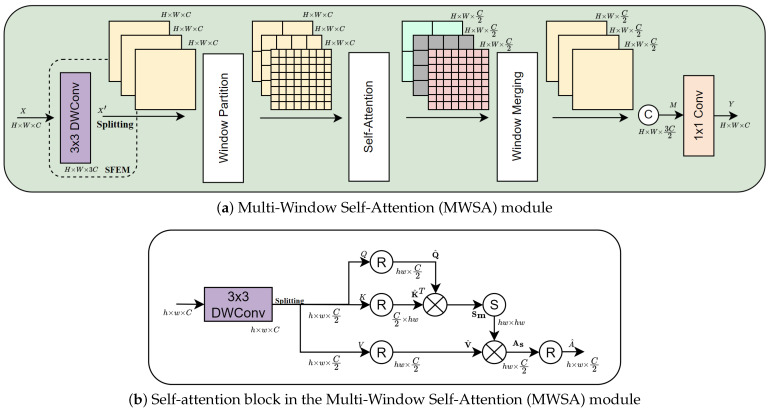
The detailed structure of MWSA.

To address the limitations of the receptive fields in previous CNNs and the fixed-size window of feature embedding in the vanilla Transformer, we designed the MWSA module, illustrated in Figure 6a. This module incorporates self-attention mechanisms across various window sizes within the spatial domain. The MWSA not only enlarges the receptive field but also enables the aggregation of multi-scale features, leading to more effective image deblurring.

Similar to MHTA, we first employ SFEM to process the input feature map $X\in R^{H\times W\times C}$. The depthwise convolution of the SFEM expands the number of channels to $X^{\prime }\in R^{H\times W\times 3C}$. Following the SFEM, the window partition module divides the three feature maps of $X^{\prime }$ (each of size $R^{H\times W\times C}$) into three feature maps containing different windows of sizes 4, 8 and 16. These partitioned windows are then passed to the self-attention block to model the structural dependencies in spatial scales. In this block, the features of each window are split into two parts producing *Q* and *V* with dimensions $R^{h\times w\times \frac{C}{2}}$, where *h* and *w* are the height and width of the partitioned windows, respectively. The key (*K*) component is set to be identical to the query (K=Q). Subsequently, a reshaping operation is applied to obtain the linear projection matrices from *Q*, *K*, and *V*. These matrices are denoted as $\hat{Q},\hat{K},\hat{V}\in R^{hw\times \frac{C}{2}}$. Finally, the attention feature is then calculated as follows:

(8) $$A_{s}=softmax\left(\frac{\hat{Q}{\hat{K}}^{T}}{\alpha }\right)\hat{V},$$

where α denotes the learnable scaling parameter used in the softmax function.

The self-attention block shown in Figure 6b is crucial for capturing multi-scale feature interactions in the MWSA module. The scaled dot-product operation is performed between the reshaped query $\left(\hat{Q}\right)$ and the reshaped transposed key $\left({\hat{K}}^{T}\right)$ matrices to obtain the spatial attention maps $S_{m}\in R^{\mathrm{hw}\times \mathrm{hw}}$. These attention maps present the compatibility scores between the pixels in windows of three different sizes (4, 8, and 16), highlighting the adaptability of this module for capturing multi-scale blur patterns. After calculating self-attention, features $A_{s}\in R^{hw\times \frac{C}{2}}$ are reshaped to the spatial dimension of the input windows resulting in features $\hat{A}\in R^{h\times w\times \frac{C}{2}}$. The window merging operation then fuses $\hat{A}$ back into the original spatial dimension and obtains three features of size $R^{H\times W\times \frac{C}{2}}$. These features represent the spatial interaction in three corresponding window sizes, which are then concatenated into feature $M\in R^{H\times W\times \frac{3C}{2}}$ to achieve complementary information from different scales. Finally, the pointwise convolution is used to transform the concatenated feature map *M* back to the input original shape and obtain the output feature $Y\in R^{H\times W\times C}$.

### 3.4. Loss Function

Loss functions are crucial for training deep-learning models [2]. However, standard loss functions typically struggle to recover the essential frequency information for generating visually clear and realistic deblurred images. In this study, we adopted a loss function that combines frequency loss [27] with absolute error loss to guide the network in restoring fine details. The overall loss function is formulated as follows:

(9) $$L_{overall}=L_{1}+\lambda L_{frequency},$$

where λ is the penalty parameter and is empirically set to 0.1.

The $L_{1}$ loss measures the pixel-wise absolute difference between the ground truth and restored images. Minimizing this term ensures that the model is faithful to the overall structure and content of the original image.

(10) $$L_{1}=\left|\right|\hat{I}-S{\left|\right|}_{1},$$

where $\hat{I}$ and *S* denote the restored and ground-truth images, respectively.

The $L_{frequency}$ loss forces the model to restore the desired frequency, resulting in a sharper deblurred image.

(11) $$L_{frequency}=||F\left(\hat{I}\right)-F\left(S\right){\left|\right|}_{1},$$

where F(·) represents the fast Fourier transform.

## 4. Experiments

This section describes the experimental setup used to assess the performance of the proposed method, including benchmark datasets, evaluation metrics, and implementation details. Subsequently, a comprehensive analysis of the proposed network is provided.

### 4.1. Dataset and Evaluation Metrics

#### 4.1.1. Dataset

We evaluated the performance of our method on synthetic as well as real-world benchmark testing datasets to analyze its generalizability and robustness. We adhered strictly to the regulations of these benchmarks to facilitate a fair comparison with other existing models. The selected benchmarks include the following:

GoPro [16]: This is a widely used dataset for evaluating image deblurring algorithms owing to its large scale. It simulates motion blurring by applying a controlled averaging process to consecutive sharp frames. Despite its convenience for controlled blur analysis, this averaging approach introduces blur artifacts that may not represent the complex blur characteristics encountered in real-world scenarios. The dataset offered 2103 blurry–sharp image pairs for training and 1111 for testing with a resolution of 1280×720.

HIDE [52]: It contains 2025 testing paired images with an image size of 1280×720. The blur was simulated via frame averaging by assigning the central frame to the ground-truth image. Although this dataset facilitates the evaluation of human-aware motion deblurring, it may not fully capture the complexities of real-world scenes, owing to the limitations of its varying blur characteristics.

RealBlur [53]: This benchmark provides a more realistic evaluation by incorporating real-world images captured using a complex camera system. It includes two versions: RealBlur-J in JPEG format and RealBlur-R in the raw format. Each version offered 3758 training and 980 testing image pairs with various image sizes.

#### 4.1.2. Evaluation Metrics

We quantitatively evaluated the performance of our deblurring method using well-known metrics on various benchmark datasets. Specifically, we calculated the average peak signal-to-noise ratio (PSNR) in decibels (dB) and the structural similarity index measure (SSIM) between the full-resolution restored images and their corresponding ground-truth images on a pixel-to-pixel basis [54]. The evaluation followed the specific protocols of each benchmark dataset. The PSNR between the ground-truth image *S* and restoration image $\hat{I}$, both of size H×W, is defined by:

(12) $$PSNR\left(S,\hat{I}\right)=10\log_{10}\left(\frac{255^{2}}{MSE(S,\hat{I})}\right),$$

where

(13) $$MSE\left(S,\hat{I}\right)=\frac{1}{HW}\sum_{i=1}^{H}\sum_{j=1}^{W}{\left(S_{ij}-{\hat{I}}_{ij}\right)}^{2}.$$

In addition, the SSIM metric [55] is used to measure the similarity between the ground-truth and restoration images, which is defined as:

(14) $$SSIM\left(S,\hat{I}\right)=\frac{\left(2\mu_{S}\mu_{\hat{I}}+C_{1}\right)\left(2\sigma_{S\hat{I}}+C_{2}\right)}{({\mu_{S}}^{2}+\mu_{\hat{I}}^{2}+C_{1})\left({\sigma_{S}}^{2}+{\sigma_{\hat{I}}}^{2}+C_{2}\right)},$$

where $\mu_{S}$ and $\mu_{\hat{I}}$ are the mean values of ground-truth and restoration images; $\sigma_{S}$ and $\sigma_{\hat{I}}$ are the standard deviation of ground-truth and restoration images; $\sigma_{S\hat{I}}$ is the covariance between the ground-truth and restoration images; $C_{1}$, $C_{2}$ are used to avoid a null denominator. The higher PNSR and SSIM values indicate a higher-quality restoration image.

Additionally, we report the computational efficiency in terms of the number of parameters (M) and the computational complexity in giga floating-point operations per second (GFLOPs). It is worth noting that the complexities of all methods were computed for a patch size of 256×256.

### 4.2. Implementation Details

Our experiments were performed using two variants of EHNet including the standard EHNet and the lightweight variant EHNet-S. These models are trained using the Adam optimizer [56] with the hyperparameters $\beta_{1}=0.9$ and $\beta_{2}=0.999$. The overall loss function $L_{overall}$ is described in Equation (9). We employ a cosine annealing learning rate scheduler [57] with an initial learning rate of $2\times 10^{-4}$ that gradually decreases to $10^{-7}$. The training process was conducted on a single NVIDIA GeForce RTX 3090 GPU using the PyTorch 2.3.1 framework. The EHNet was initially trained on the GoPro dataset, each training image was cropped into eight overlapping patches and augmented with random horizontal and vertical flips to improve robustness. The number of channels was set to 48 at the first scale. The numbers of encoder and decoder blocks were configured with patterns of [6,6,6] and [10,6,6] from scale levels 1 to 3, respectively. Furthermore, we applied a progressive learning strategy [28] over 500 epochs to promote model robustness, which started the training with a patch size of 256×256 and a batch size of 2. It then increases the patch size to 320×320 and decreases the batch size to 1 at the epoch 300th. Finally, the network was evaluated using the GoPro, HIDE, RealBlur-J, and RealBlur-R benchmarks.

A previous study [53] revealed that the blur characteristics in the GoPro dataset differ significantly from those encountered in real-world scenarios. Consequently, deep-learning methods trained on the GoPro dataset struggle to handle real-world blurry images effectively. EHNet was retrained on the RealBlur-J and RealBlur-R to tackle this issue, followed by benchmark evaluation.

In addition, we introduce the lightweight variant EHNet-S, which builds upon the core architecture of EHNet but adopts a compact design strategy. In particular, we halved the initial channels from 48 to 24, and used a compact configured number of blocks in the encoder and decoder as [4,4,4] and [4,4,4], respectively, from scale levels 1 to 3. The configuration retains the core functionality of EHNet with a reduced number of parameters and complexity. Similar to EHNet, EHNet-S was trained on the GoPro dataset, and a progressive learning strategy was applied over 400 epochs. The training began with a patch size of 256×256 and a batch size of 2. At the epoch 300th, the patch size increased to 384×384, whereas the batch size decreased to 1.

### 4.3. Performance Comparison

This section evaluates EHNet by comparing it with state-of-the-art methods in both quantitative and qualitative aspects using synthetic as well as real-world image deblurring datasets. Additionally, we compared EHNet-S with lightweight deblurring methods to investigate the effectiveness of the proposed method.

#### 4.3.1. EHNet

We trained EHNet on the GoPro training set and compared it with nine leading deep-learning-based image deblurring methods such as DeblurGAN-v2 [19], DBGAN [20], MIMO-UNet+ [27], MPRNet [28], HINet [29], Restormer [37], BANet+ [31], Stripformer [41], and FSNet [34]. To ensure a fair evaluation, we utilized pre-trained models on the GoPro training set and quantitative results from the reports provided by the authors of these studies. All the methods were evaluated on the benchmark datasets GoPro [16], HIDE [52], RealBlur-J [53], and RealBlur-R [53] with full-resolution images for testing.

Table 1 presents the performance of EHNet trained on the GoPro dataset. It achieved the highest PSNR and SSIM scores on the GoPro and HIDE datasets and maintained the second-highest SSIM and third-highest PSNR on the RealBlur-R dataset. It is noteworthy that EHNet accomplishes these results with the lowest number of parameters and computational complexity among the compared methods. Specifically, our model utilizes only 8.78 million parameters and 91 GFLOPs. The main idea of our paper is to optimize the deblurring performance related to the number of parameters and complexity of the model. Our approach enhances performance while reducing both parameters and complexity by combining efficient CNN modules with Transformer modules to address their inherent limitations in their feature maps. The proposed modules are designed with that trade-off between small performance and significant model size and complexity. We free up computational resources by adopting these efficient components; this manner allows the model to increase the number of channels in the feature maps of the encoder and decoder for improving deblurring performance. The competitive performance and resource efficiency make EHNet suitable for applications with constrained computational capabilities.

**Figure 7:**
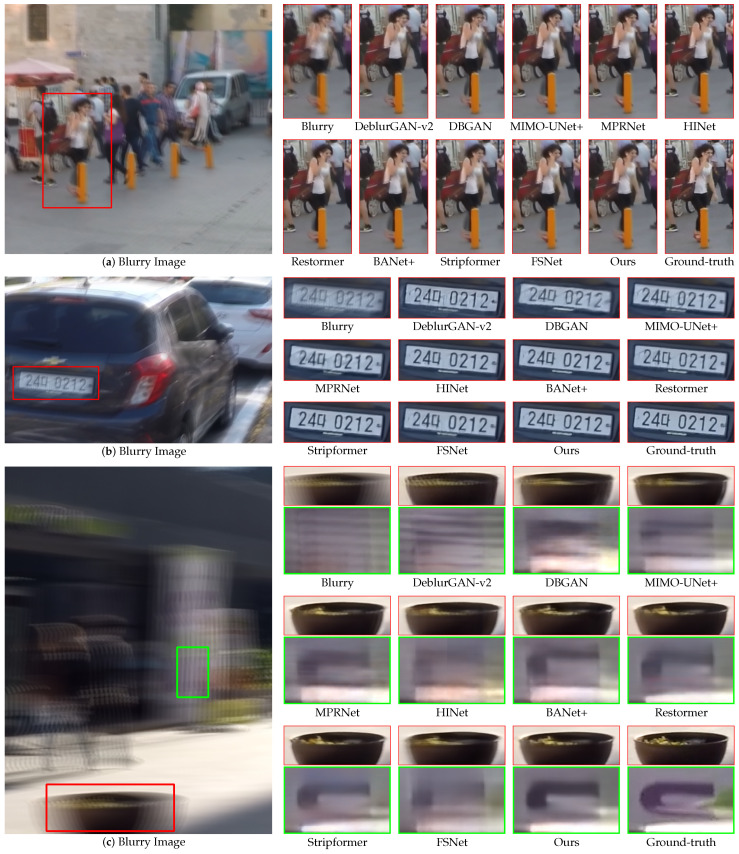
Qualitative comparisons of zoomed-in patches on the GoPro dataset.

**Figure 8:**
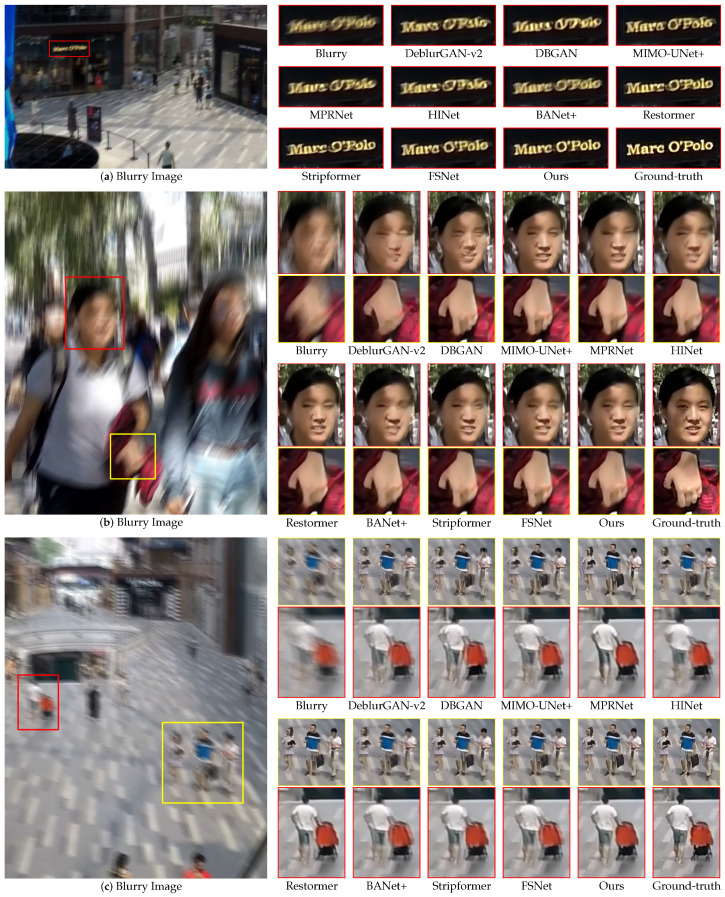
Qualitative comparisons of zoomed-in patches on the HIDE dataset.

Figure 7 and Figure 8 present qualitative comparisons of the deblurring methods trained on the GoPro dataset and evaluated using the GoPro and HIDE test sets. Our proposed EHNet demonstrated superiority in restoring texture and detail with diverse backgrounds and blur characteristics.

Figure 7a evaluates the deblurring results of comparison methods in handling small details. For instance, DeblurGAN-v2, DBGAN, MPRNet, HINet, Restormer, BANet+, and Stripformer face challenges in recovering the left arm of a woman within a red bounding box. By contrast, MIMO-UNet+, FSNet, and our EHNet model successfully preserved this detail. Furthermore, only EHNet can recover the shape of the right leg of the woman. Figure 7b shows a scenario involving blurry text on a car plate. DeblurGAN-v2, DBGAN, MIMO-UNet+, MPRNet, HINet, Restormer, BANet+, and FSNet exhibit limited text recovery. However, Stripformer and EHNet effectively restore clear details and text edges. Figure 7c illustrates a scene with severe blurring within the red bounding box, where DeblurGAN-v2, DBGAN, MIMO-UNet+, MPRNet, HINet, and FSNet fail to recover the edge details of pots and leaves. Conversely, BANet+, Restormer, Stripformer, and EHNet achieve superior detailed restorations. Moreover, the green bounding box highlights the challenging region where only EHNet accurately reconstructs the U-shaped text panel, presenting its ability to recover complex structures in heavily blurred scenes. In summary, these visual comparisons with many types of blur in images show that EHNet restored details clearer than the comparison methods on the GoPro test set.

Figure 8a depicts the deblurred text panel in a distant scene. DeblurGAN-v2, DBGAN, MIMO-UNet+, MPRNet, HINet, BANet+, and Stripformer struggle to restore the shape and edges of the text. By contrast, Restormer, FSNet, and EHNet successfully recovered the text shape. Notably, EHNet restores the primary shapes of the text and eliminates unwanted artifacts. This result shows the ability of EHNet to recover fine details while preserving the overall structure of objects. Figure 8b shows a blurry scene of individuals in a crowd. DeblurGAN-v2, DBGAN, MPRNet, HINet, BANet+, Stripformer, and FSNet struggle to handle details in the faces of the women. By contrast, MIMO-UNet+, Restormer, and EHNet can restore facial detail. Almost all methods fail to recover the shape of the fingers, whereas MIMO-UNet+, FSNet, and our EHNet can recover them. It is important to note that all models were trained on the GoPro training set using 256×256 patches but are evaluated on full-resolution images (e.g., 1280×720 in the GoPro and HIDE datasets). This discrepancy presents a significant challenge in deblurring fine object details. Figure 8c illustrates the deblurring of distant and small pedestrians. DeblurGAN-v2, DBGAN, MIMO-UNet+, HINet, BANet+, Stripformer, and FSNet struggle to remove the blur. By contrast, MPRNet, Restormer, and our proposed EHNet restore details of the human and black bags and preserve the overall structure of the restoration images. These qualitative results further underscore the effectiveness of EHNet in restoring fine details across diverse blur types, demonstrating its superior performance compared with several state-of-the-art deep-learning methods.

Although its prevalence in image deblurring research, the GoPro dataset presents limitations in training generalized models. The dataset is synthesized using a frame-averaging method resulting in unrealistic and discontinuous blur characteristics. Consequently, data-driven approaches trained on GoPro often fail to capture the diverse blur patterns encountered in real-world scenarios [53]. This poses a significant challenge for deep-learning methods, which are susceptible to variations in training data across different environments.

To address this limitation and conduct a more comprehensive evaluation, we followed a protocol established using the RealBlur dataset. We trained EHNet on the RealBlur-J and RealBlur-R training sets, which offer a more realistic representation of real-world blur variations. This approach allowed our trained model to be fairly compared with the pre-trained models of recent methods on these datasets including SRN [17], DeblurGAN-v2 [19], MSSNet [30], Stripformer [41], and BANet+ [31]. In summary, this evaluation provides insights into the ability of each method to handle real-world blur variations.

**Figure 9:**
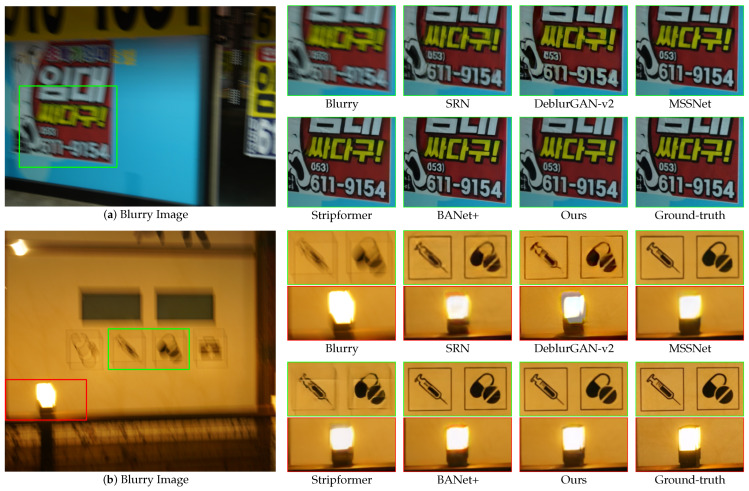
Qualitative comparisons of zoomed-in patches on the RealBlur-J dataset.

**Figure 10:**
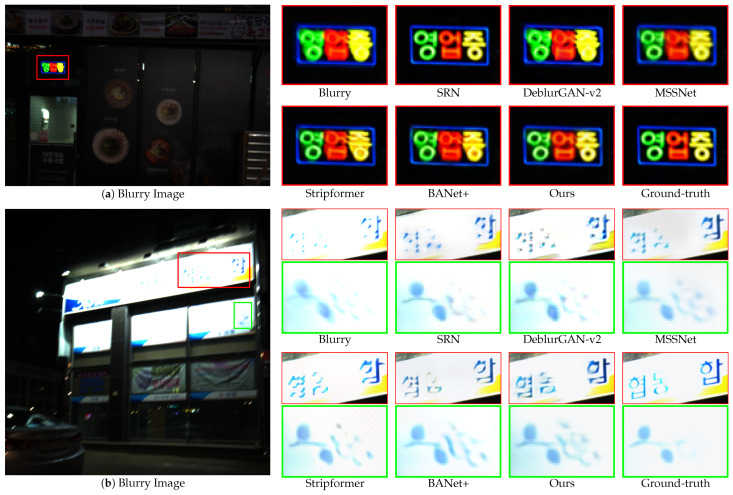
Qualitative comparisons of zoomed-in patches on the RealBlur-R dataset.

Table 2 indicates that our model achieved outstanding quantitative results for both the PSNR and SSIM metrics on RealBlur-J and RealBlur-R datasets. This performance is particularly noteworthy given the compact model size and reduced computational complexity of EHNet compared with other methods. Figure 9 and Figure 10 provide visual comparisons with recent methods for the RealBlur-J and RealBlur-R datasets, respectively. These qualitative and quantitative assessments offer a comprehensive evaluation of the performance of EHNet in real-world scenarios.

Figure 9a presents a scene with blurry text in a real-world image in the RealBlur-J test set. SRN, DeblurGAN-v2, MSSNet, and Stripformer exhibit limitations in terms of recovering text clarity. Conversely, BANet+ and EHNet successfully restored the shape of the text and eliminated the residual blur. Figure 9b highlights the challenges posed by complex blur patterns. In the green bounding box, SRN, DeblurGAN-v2, and Stripformer failed to recover the shapes of the wall pictures. Although Stripformer offers favorable results in overall image deblurring, its reliance on horizontal and vertical strip attention can hinder detailed recovery and introduce artifacts, particularly when dealing with horizontal and vertical blur patterns. This limitation leads to a failure to restore the shape in some cases. In contrast, MSSNet, BANet+, and our EHNet can recover the original shapes of the wall pictures. Especially, in the red bounding box of Figure 9b, only EHNet restores the fine details of the lantern without distorting the light source. Experiments on the Realblur-J dataset show that EHNet outperforms other methods in restoring details, demonstrating its ability to handle complex patterns and light distortion conditions in real-world scenarios.

Figure 10a depicts a real-world dark scene with blurry-colored text. SRN, DeblurGAN-v2, MSSNet, Stripformer, and BANet+ struggle to recover text without spatial color distortion. In contrast, EHNet excels at restoring text with minimal spatial color distortion. Figure 10b presents a challenging scenario with a saturated area, where the details and shapes are nearly obscured. The effectiveness of EHNet in restoring the desired details and textures is evident in this context. These qualitative results indicate that our model has a strong capacity for deblurring in real-world images with low-light blur conditions.

#### 4.3.2. EHNet-S

In addition to the standard EHNet architecture, we introduce a lightweight variant EHNet-S, which maintains the core functionality of EHNet while requiring fewer parameters and computations. EHNet-S was then compared to several lightweight deblurring models, including PSS-NSC [21], MT-RNN [22], SAPHN [24], MIMO-UNet [27], and CODE [38]. To ensure a fair evaluation, all the methods were trained on the GoPro training set and subsequently tested on its corresponding test set.

The quantitative evaluation results in Table 3 show that EHNet-S surpasses the other lightweight deblurring methods. Remarkably, EHNet-S achieved the highest PSNR and SSIM scores while maintaining the smallest number of parameters and computational complexity. The good trade-off between performance and complexity proves that EHNet-S is a well-suited solution for image deblurring applications with limited computational resources.

### 4.4. Ablation Studies

#### 4.4.1. Effectiveness of MHTA and MWSA

This section validates the effectiveness of the MWSA and MHTA in the EHNet-S model on the GoPro testing set in terms of performance, memory usage, and computational cost. We systematically re-trained the four model variations including or excluding these attention modules, as shown in Table 4. The results show that Model II using MWSA outperforms Model I using MHTA by 0.48 dB for PSNR and 0.004 for SSIM with a gain of 0.03 M in the number of parameters and 0.37 GFLOPs. Furthermore, the EHNet-S incorporating both MHTA and MWSA modules achieved higher performance with a slight increase in memory and computational cost. Specifically, EHNet-S gains 0.44 dB in PSNR and 0.005 in SSIM, and 0.1 M parameters, 1.51 GFLOPs to Model II.

**Figure 11:**
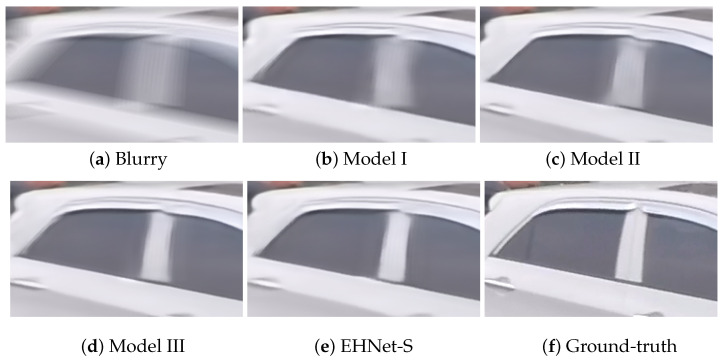
Visualization of the effectiveness of proposed modules.

Figure 11 visually compares the deblurring results of these models and shows that EHNet-S recovers clearer object textures than its counterparts. This experiment highlights the effect of combining MWSA and MHTA to enhance deblurring performance.

#### 4.4.2. Effectiveness of SFEM

This section investigates the impact of integrating the SFEM into the overall network. As shown in Table 5, EHNet-S achieves a PSNR of 32.04 dB, reflecting a slight performance decrease of 0.05 dB compared to Model III, which uses pointwise and depthwise convolutions to generate embedding features in the self-attention mechanism. Nonetheless, incorporating SFEM significantly reduces the model size by 0.45 MB (approximately 22%) and the computational complexity by 6.23 GFLOPs (approximately 27%). These findings underscore the potential of the SFEM to optimize the efficiency of the Transformer while maintaining deblurring performance, making it a promising method for resource-constrained applications.

## 5. Conclusions

This study introduced a hybrid deep-learning architecture called EHNet for efficient image deblurring. By integrating the strengths of CNNs for local feature extraction and Transformers for capturing long-range dependencies, EHNet overcomes the limitations of existing models. Furthermore, it enhances the vanilla Transformer by incorporating attention mechanisms in both channel (MHTA) and spatial (MWSA) domains to capture channel interactions and multi-scale long-range dependencies. In addition, we introduce the SFEM to mitigate the high computational complexity of Transformers, thereby EHNet is appropriate for deployment in resource-limited environments. The experimental results demonstrate that EHNet performs favorably against state-of-the-art deep-learning deblurring methods on synthetic as well as real-world benchmarks while maintaining a compact model size and complexity.

Deep-learning models hold significant potential for image deblurring, but their performance relies on the training dataset. Blurry images can originate from diverse sources, including synthetically generated data, such as those produced by applying blur kernels or frame averaging techniques, as well as real-world scenarios involving camera shake or object motion. This inherent variability in the blur characteristics impedes the generalization ability of deep models when handling unseen datasets. Future research should prioritize reducing dependency on the training data dependency to enhance the adaptability of deep-learning models. A promising direction is to investigate techniques for transferring the characteristics of unseen blurry images to the blur characteristics used for training deblurring models. This approach could enable the deep-learning-based deblurring models to handle unseen blurry images without additional training on new datasets, thereby improving the robustness of these models.

## Figures and Tables

**Table 1:** Quantitative comparison on several benchmarks with state-of-the-art deep-learning methods trained on the GoPro dataset. The red, blue, and green denote the best results, second-best and the third-best results, respectively. The symbol “↑” indicates higher is better and “↓” means that lower is better.

Methods	GoPro [16]		HIDE [52]		RealBlur-J [53]		RealBlur-R [53]		Params↓ (M)	Complexity↓ (GFLOPs)
	PSNR↑	SSIM↑	PSNR↑	SSIM↑	PSNR↑	SSIM↑	PSNR↑	SSIM↑		
DeblurGAN-v2 [19]	29.55	0.934	26.61	0.875	**28.70**	0.866	35.26	0.944	60.9	411
DBGAN [20]	31.10	0.942	28.94	0.915	24.93	0.745	33.78	0.909	**11.6**	760
MIMO-UNet+ [27]	32.45	0.958	29.99	0.930	27.63	0.837	35.54	0.947	16.1	145
MPRNet [28]	32.66	0.959	30.96	0.939	**28.70**	**0.873**	35.99	0.952	20.1	778
HINet [29]	32.71	0.959	30.32	0.932	28.17	0.849	35.75	0.950	88.7	171
Restormer [37]	32.92	0.961	**31.22**	**0.942**	**28.96**	**0.879**	**36.19**	**0.957**	26.1	**140**
BANet+ [31]	33.03	0.961	30.58	0.935	28.10	0.852	35.78	0.950	40.0	588
Stripformer [41]	**33.08**	**0.962**	31.03	0.939	**28.82**	**0.876**	**36.08**	**0.954**	19.7	170
FSNet [34]	**33.28**	**0.963**	**31.05**	**0.941**	28.47	0.868	35.84	**0.952**	**13.28**	**111**
**EHNet**	**33.32**	**0.964**	**31.34**	**0.943**	28.22	0.854	**36.06**	**0.954**	**8.78**	**91**

**Table 2:** Performance comparison on RealBlur testing sets with methods trained on the RealBlur training sets. The **bold** text indicates the best results, the symbol “↑” indicates that higher is better, and “↓” means that lower is better.

Methods	RealBlur-J [53]		RealBlur-R [53]		Params↓ (M)	Complexity↓ (GFLOPs)
	PSNR↑	SSIM↑	PSNR↑	SSIM↑		
SRN [17]	31.38	0.909	38.65	0.965	**6.8**	167
DeblurGAN-v2 [19]	29.69	0.870	36.44	0.935	60.9	411
MSSNet [30]	32.10	0.928	39.76	0.972	15.6	154
Stripformer [41]	32.48	0.929	39.84	**0.974**	19.7	170
BANet+ [31]	32.40	0.929	39.90	0.972	40.0	588
**EHNet**	**32.50**	**0.931**	**40.06**	**0.974**	8.78	**91**

**Table 3:** Quantitative comparison of EHNet-S on the GoPro dataset. The **bold** text highlights the best results, the symbol “↑” indicates that higher is better, and “↓” means that lower is better. The symbol “-” denotes the unknown values. Additionally, the symbol “†” denotes works that did not release the code or pre-trained weights; results for these studies are sources from the original papers.

Methods	GoPro [16]		Params↓ (M)	Complexity↓ (GFLOPs)
	PSNR↑	SSIM↑		
PSS-NSC [21]	30.92	0.935	2.84	29.11
MT-RNN [22]	31.15	0.945	2.64	164.00
MIMO-UNet [27]	31.73	0.951	6.80	67.17
SAPHN † [24]	31.85	0.948	23.00	-
CODE † [38]	31.94	-	12.18	22.52
**EHNet-S**	**32.04**	**0.954**	**1.58**	**16.85**

**Table 4:** Ablation experiments for attention modules in Transformer Blocks. The results are evaluated on the GoPro test set. The symbol “↑” indicates that higher is better, and “↓” means that lower is better. Additionally, the symbols “✓” and “✗” denote the presence and absence of modules, respectively.

Model	MHTA	MWSA	PSNR↑	SSIM↑	Params↓ (M)	Complexity↓ (GFLOPs)
I	✓	✗	31.12	0.945	1.45	14.97
II	✗	✓	31.60	0.949	1.48	15.34
EHNet-S	✓	✓	32.04	0.954	1.58	16.85

**Table 5:** The effectiveness of SFEM on the EHNet-S. “PW+DW” denotes the model employs pointwise and depthwise convolutions to generate embedding features. The symbol “↑” indicates that higher is better, and “↓” means that lower is better. Additionally, the symbols “✓” and “✗” denote the presence and absence of modules, respectively.

Model	PW + DW	SFEM	PSNR↑	SSIM↑	Params↓ (M)	Complexity↓ (GFLOPs)
III	✓	✗	32.09	0.954	2.03	23.08
EHNet-S	✗	✓	32.04	0.954	1.58	16.85

## Data Availability

Data are contained within the article. Anyone can use or modify the source code for only academic purposes. The experiment results, source code, and pre-trained models are available at https://github.com/hoquocthien/EHNet-Efficient-Hybrid-Network-with-Dual-Attention-for-Image-Deblurring (accessed on 7 October 2024).
